# Association between healthy lifestyle factors and health-related quality of life among Chinese adolescents: the moderating role of gender

**DOI:** 10.1186/s12955-023-02201-2

**Published:** 2023-10-31

**Authors:** Hongyu Xiang, Xiuqiong Feng, Li Lin, Shengyu Luo, Xinxia Liu, Dezhong Chen, Kang Qin, Xun Guo, Weiqing Chen, Vivian Yawei Guo

**Affiliations:** 1 Department of Epidemiology, School of Public Health, Sun Yat-sen University, Guangzhou, Guangdong 510080 China; 2 Department of Public Health, Guangzhou Huangpu District Center for Disease Control and Prevention, Guangzhou, Guangdong 510530 China; 3 Zhongshan Third People’s Hospital, Nanlang Town, Zhongshan, Guangdong 528451 China

**Keywords:** Healthy lifestyle, Health-related quality of life, Adolescent, Moderation, Gender difference

## Abstract

**Background:**

To examine the associations of the independent and combined healthy lifestyle factors with health-related quality of life (HRQOL) in adolescents, and to test the moderating role of gender.

**Methods:**

This cross-sectional study included 5125 adolescents aged between 11 and 20 years. They provided self-reported data on six healthy lifestyle factors, including never smoking, never drinking, good sleep quality, sufficient sleep duration, appropriate Internet use, and adequate physical activity. Adolescents’ HRQOL was evaluated using the Pediatric Quality of Life Inventory version 4.0. Linear regression models were conducted to explore the association of individual and combined healthy lifestyle factors with adolescents’ HRQOL. We further performed stratified analyses and likelihood ratio test to explore the moderating role of gender in these associations.

**Results:**

Of the included adolescents, the proportions with 0–2, 3, 4, and 5–6 healthy lifestyle factors were 13.6%, 26.4%, 44.3%, and 15.7%, respectively. Compared to adolescents with composite healthy lifestyle scores of 0–2, those with scores of 3, 4, or 5–6 had significantly higher HRQOL scores across all dimensions, summary scales, and total scale in both unadjusted and adjusted models. Specifically, adolescents with 5–6 healthy lifestyle factors had a total scale score that was 19.03 (95%CI: 17.76 to 20.30) points higher than their counterparts who only had 0–2 healthy lifestyle factors. Significant dose-response patterns were also observed in aforementioned associations. Gender was a significant moderator in the associations between composite healthy lifestyle groups and HRQOL scores, except for the social functioning dimension.

**Conclusions:**

Our results confirmed that combined healthy lifestyle factors were associated with improved HRQOL among adolescents, with a stronger association observed in girls. These findings underscore the necessity for education and healthcare authorities to design health-promoting strategies that encourage multiple healthy lifestyle factors in adolescents, with the objective of enhancing their overall health outcomes.

## Introduction

Health-related quality of life (HRQOL) is a subjective measure that could reflect an individual’s overall health, encompassing physical, mental, and social well-being [1]. It is widely used in clinical practice to assess and monitor patients’ well-being, thereby informing treatment decisions [1–4]. In recent years, HRQOL has been applied to the general population [5–7]. Previous studies have found that lower HRQOL scores were associated with increased risks of hospitalization and even mortality [8–10]. HRQOL has also been integrated into clinical practice and public health research for children and adolescents [11].

Various factors, such as age, gender, socioeconomic status, and the presence of chronic diseases, can influence HRQOL [12–14]. There is also evidence suggesting that healthy lifestyle factors, e.g., abstaining from smoking and drinking, maintaining good sleep quality and sufficient sleep duration, appropriate Internet use, and engaging in adequate physical activity, are associated with higher HRQOL scores in children and adolescents [15–20]. For instance, a cross-sectional study has found that non-smoking adolescents reported better self-rated health and life satisfaction compared to active or passive smokers [15]. Another randomized controlled trial has shown that adolescents who received interventions to reduce binge drinking had higher HRQOL scores than those in the control group [16]. Similar positive associations have also been found between good sleep health and better HRQOL in adolescents [17, 18]. In addition, a cross-sectional study has revealed that adolescents with adaptive Internet use exhibited fewer depressive symptoms and higher HRQOL scores than those with maladaptive and pathological Internet use [19]. Furthermore, a previous study has found a positive association between higher level of physical activity and better HRQOL among Chinese adolescents [20]. While many studies have addressed the impact of individual lifestyle factors on HRQOL, research has suggested that these lifestyle factors often co-occur [21] and are cumulatively linked to health outcomes [22, 23]. Consequently, there is a need to further explore the influence of cumulative lifestyle factors on HRQOL in adolescents, in order to inform early interventive strategies. However, limited research conducted in Europe and Australia have explored this issue in adolescents [24–26]. Since lifestyle patterns and self-rated HRQOL vary among adolescents from different countries due to cultural and socioeconomic differences [27–29], it is therefore crucial to investigate the associations between cumulative healthy lifestyle factors and HRQOL in the context of Chinese culture.

In addition, the role of gender in the associations between healthy lifestyle factors and health outcomes remain inconsistent [26, 30]. A longitudinal study conducted in Australia has revealed that compared to adolescents with 0 or 1 healthy lifestyle factor, those reporting 4 or 5 healthy lifestyle factors had significantly higher scores of physical HRQOL in both boys and girls [26]. However, such impact on the total HRQOL scores was only significant in boys, but not in girls [26]. Furthermore, a meta-analysis of 38 articles has found no gender difference in the association between physical activity, sedentary behavior, and self-rated health among children and adolescents [30]. These inconsistent findings may result from the heterogeneous healthy lifestyle factors and HRQOL measures that were investigated, highlighting the need for further research to clarify the role of gender in the association between combined healthy lifestyle factors and HRQOL in Chinese adolescents.

Therefore, in this study, we aimed to explore the association of a composite healthy lifestyle score that was generated based on smoking status, drinking status, sleep quality, sleep duration, Internet addiction, and physical activity, with HRQOL in adolescents attending middle schools. Stratified analyses and tests for interaction were further conducted to evaluate the potential moderating role of gender in this association.

## Methods

### Study design and population

This cross-sectional study was conducted between November and December 2021 and recruited middle school students from the Huangpu district of Guangzhou, China. In order to obtain a representative study sample, we randomly selected six middle schools, proportional to the district’s total number of middle schools. The selection comprised four junior middle schools and two combined junior and senior middle schools. We invited all students from these schools, along with their parents, to participate in the study. Out of the invited participants, 6982 adolescents, including 4330 junior middle school students and 2652 senior middle school students, with parental consent had completed the questionnaires, yielding a 90.1% response rate. We then excluded 1729 adolescents due to incomplete information on healthy lifestyle factors and 128 adolescents with missing data on HRQOL. Ultimately, a total of 5125 adolescents were included in the current analysis.

The present research project was approved by the Ethics Committee of School of Public Health, Sun Yat-sen University (Reference number: 2021[116]). Prior to the survey, parents of each student have signed an informed consent for their children to participate in this study.

### Assessment of health-related quality of life

Adolescents’ HRQOL was evaluated using the Pediatric Quality of Life Inventory version 4.0 (PedsQL 4.0). This multidimensional instrument consists of 23 items that evaluate emotional functioning (5 items), physical functioning (8 items), social functioning (5 items), and school functioning (5 items) [31–34]. The reliability and validity of PedsQL 4.0 have been confirmed among Chinese children and adolescents [35]. Each item was rated based on a five-point Likert scale ranging from 0 to 4 (0 = never a problem, 1 = almost never a problem, 2 = sometimes a problem, 3 = often a problem, and 4 = almost always a problem). To calculate HRQOL scores, responses were reversely scored and linearly transformed into a 0 to 100 scale, where higher scores represented better HRQOL. The score for each dimension was calculated by averaging the scores of all items within that dimension. In addition, the scores of the psychosocial health summary scale were calculated as the average scores of the emotional, social, and school functioning dimensions. The total scale scores were further computed as the average scores of all items in the questionnaire.

### Assessment of composite healthy lifestyle score

Smoking and drinking status were divided into ever and never users by asking the questions “Have you ever smoked an entire cigarette?” and “Have you ever had a glass of wine/beer?”, respectively.

Sleep quality and duration were assessed by the Pittsburgh Sleep Quality Index (PSQI) questionnaire [36]. which has been validated and proven to be reliable in Chinese children and adolescents [37]. The questionnaire measures seven different sleep dimensions, including subjective sleep quality, sleep latency, sleep duration, habitual sleep efficiency, sleep disturbance, use of sleep medications, and daytime dysfunction. A global PSQI score ranging from 0 to 21 was calculated by summing scores of the seven dimensions, with higher scores indicating poorer sleep quality. We defined good sleep quality as a PSQI global score ≤ 7 in Chinese version [37]. Sufficient sleep duration was determined using cutoff values of 9 h per night for junior middle school students and 8 h per night for senior middle school students, according to the Chinese guideline [38].

The Young Diagnostic Questionnaire (YDQ) was used to assess the addiction level of Internet use [39], a valid and reliable instrument in Chinese young population [40]. The questionnaire consists of eight items regarding Internet addiction, such as “feeling preoccupied with the Internet”, “feeling the need to use the Internet for an increasing amount of time”, and “unsuccessful efforts to control Internet use”. Each of the item was dichotomized as either yes (coded as 1) or no (coded as 0). The cumulative YDQ score was calculated by adding up the eight items, with higher scores representing a higher level of Internet addiction. We defined the group with appropriate Internet use as those with a YDQ score < 5 and the group with Internet addiction as those with a score of 5 or above [39].

To assess physical activity, the validated Chinese version of the International Physical Activity Questionnaire-Short Form (IPAQ-SF) was used [41]. It covers questions related to the frequency and duration of vigorous, moderate, and light physical activity, as well as the daily duration of sedentary behavior, in the past seven days. We defined adequate physical activity as having moderate or vigorous physical activity for at least 60 min per day and having at least 10 min vigorous physical activity for more than three days per week according to the Physical Activity Guidelines for Chinese adolescents [42].

We assigned 1 point for each of the aforementioned healthy lifestyle factors: i.e., never smoking, never drinking, good sleep quality, sufficient sleep duration, appropriate Internet use, and adequate physical activity. A composite healthy lifestyle score (range: 0–6 points) was generated by summing the points for these six healthy lifestyle factors, with higher scores representing a healthier lifestyle. The composite healthy lifestyle scores were further reclassified into four groups according to the distribution among the participants: (1) 0–2 healthy lifestyle factors, (2) 3 healthy lifestyle factors, (3) 4 healthy lifestyle factors, and (4) 5–6 healthy lifestyle factors.

### Covariates

#### Adolescents

Adolescents provided self-reported information on their age, gender, single child status, primary caregiver, and boarding school attendance. Single child status was identified as having only one child in the family (yes) or having more than one child in the family (no). Primary caregivers were categorized as either parents or others. Boarding school attendance was dichotomized as attending a boarding school (yes) and not attending a boarding school (no).

#### Parents

Parental socio-demographic characteristics, including age, marital status, and occupational status, were also collected. Parents self-reported their age and occupational status through online questionnaire. Occupational status was classified as employed or unemployed. Marital status was reported by their children, which was categorized as currently married or unmarried. The latter included single, divorced, separated, and widowed.

### Statistical analysis

Descriptive statistics for continuous and categorical variables were presented as means with standard deviation (SD) and frequencies with percentage, respectively. To compare differences in characteristics across the composite healthy lifestyle groups, one-way analysis of variance (ANOVA) was used for continuous data and Chi-squared test was applied for categorical data. To assess trends in characteristics across different composite healthy lifestyle groups, polynomial comparisons were used for continuous data and Mantel-Haenszel statistic was applied for categorical data.

Linear regression models were established to assess the associations of both individual and combined healthy lifestyle factors with HRQOL. Crude models were first constructed. Then, adjusted models were further established with adjustment for adolescents’ age, gender, single child status, primary caregiver, and boarding school attendance, as well as parental age, marital status, and occupational status. When the exposure was the individual healthy lifestyle factor, models were further mutually adjusted for other healthy lifestyle factors. When the exposure was the composite healthy lifestyle groups, dose-response associations with adolescents’ HRQOL were assessed with trend tests. The linear regression assumptions of linearity, normality, homoscedasticity, and absence of multicollinearity were verified for all models. Results were reported as beta coefficient (*β*) with corresponding 95% confidence intervals (CI). Stratified analyses were further conducted by gender. The potential moderating role of gender in the association between healthy lifestyle scores and HRQOL was assessed by likelihood ratio test.

All data analyses were performed with Stata/SE 17.0. Statistical significance was two-sided with a *P* value < 0.05.

## Results

**Fig. 1:**
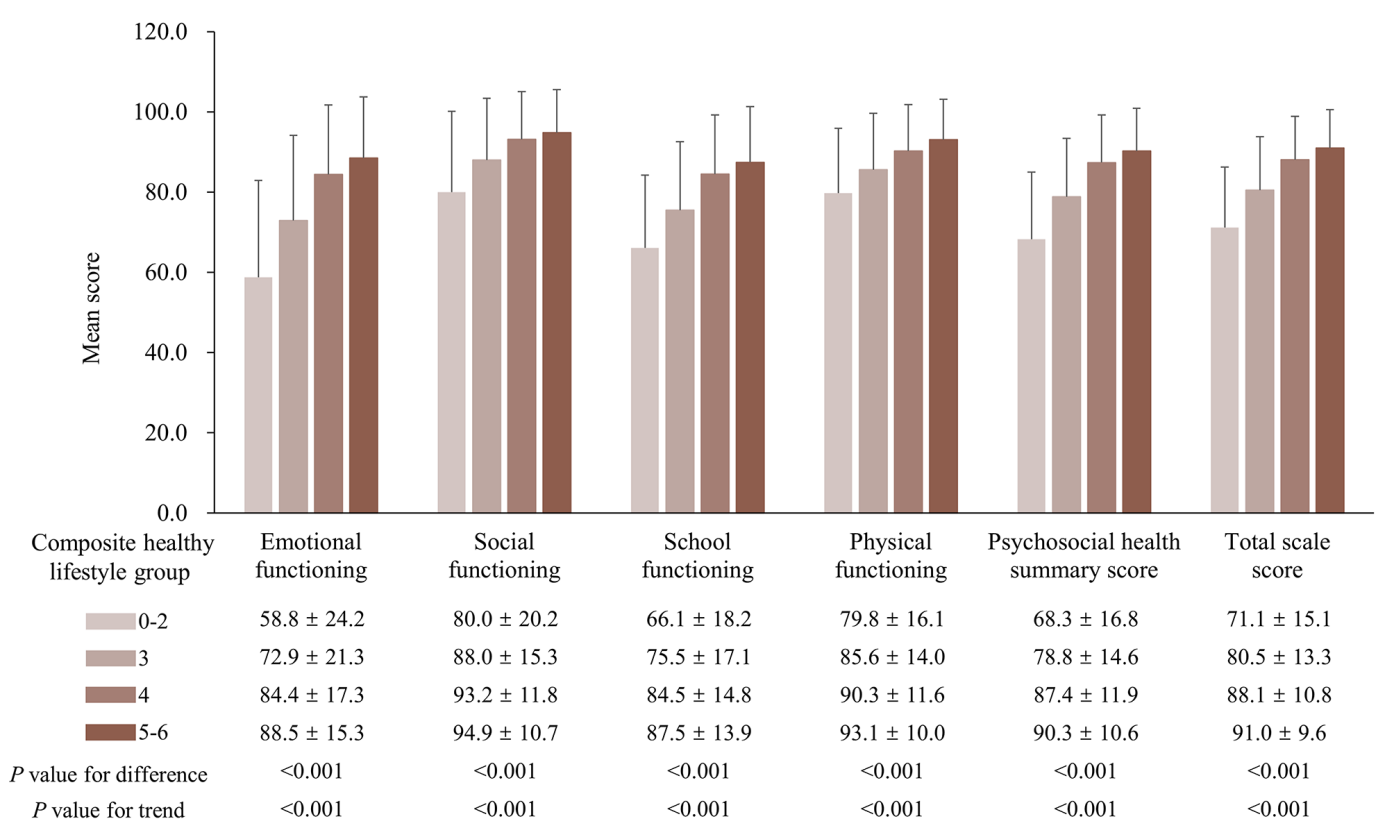
Comparison of adolescents’ HRQOL across the composite healthy lifestyle groups

Of the 5125 adolescents included in the study, 2690 (52.5%) were boys and the average age was 14.6 (SD: 1.6) years. The prevalence of healthy lifestyle factors was 97.3% for never smoking, 69.4% for never drinking, 82.0% for good sleep quality, 12.6% for sufficient sleep duration, 83.2% for appropriate Internet use, and 15.3% for adequate physical activity. In terms of the combined healthy lifestyle factors, the proportions of adolescents with 0–2, 3, 4, and 5–6 healthy lifestyle factors were 13.6%, 26.4%, 44.3%, and 15.7%, respectively. In general, compared to adolescents with 0–2 healthy lifestyle factors, those with a composite healthy lifestyle score of 5–6 tended to be younger, were more likely to be boys, have parents as their primary caregivers, and not attend boarding schools. The parents of adolescents with a composite healthy lifestyle score of 5–6 were also more likely to be younger and married compared to the group with a composite healthy lifestyle score of 0–2 (Table 1). Furthermore, as the composite healthy lifestyle score increased, the HRQOL scores across all HRQOL dimensions, summary scales, and total scale were significantly higher (Fig. 1).

**Table 1:** Comparison of characteristics across the composite healthy lifestyle groups in adolescents

		Composite healthy lifestyle score				*P* value for difference	*P* value for trend
Characteristics		0–2 n = 699	3 n = 1353	4 n = 2271	5–6 n = 802		
**Adolescents**							
Age (years), mean (SD)		15.2 (1.5)	15.0 (1.6)	14.3 (1.6)	14.0 (1.5)	< 0.001	< 0.001
Gender, n (%)						< 0.001	< 0.001
	Boy	343 (49.1%)	687 (50.8%)	1148 (50.6%)	512 (63.8%)		
	Girl	356 (50.9%)	666 (49.2%)	1123 (49.4%)	290 (36.2%)		
Single child status, n (%)						0.320	0.317
	Yes	200 (28.8%)	389 (28.9%)	595 (26.4%)	224 (28.2%)		
	No	494 (71.2%)	956 (71.1%)	1661 (73.6%)	571 (71.8%)		
Primary caregiver, n (%)							
	Parents	640 (93.2%)	1269 (95.6%)	2145 (96.1%)	765 (96.1%)	0.009	0.006
	Others	47 (6.8%)	58 (4.4%)	87 (3.9%)	31 (3.9%)		
Boarding school attendance, n (%)						< 0.001	< 0.001
	Yes	518 (75.0%)	1004 (75.0%)	1503 (67.1%)	460 (58.5%)		
	No	173 (25.0%)	334 (25.0%)	738 (32.9%)	326 (41.5%)		
**Parents**							
Maternal age (years), mean (SD)		42.1 (4.5)	41.9 (4.2)	41.3 (4.3)	40.6 (4.6)	< 0.001	< 0.001
Paternal age (years), mean (SD)		44.7 (4.8)	44.6 (4.6)	43.8 (4.7)	42.9 (4.7)	< 0.001	< 0.001
Marital status, n (%)						0.021	0.003
	Married	656 (93.8%)	1276 (94.3%)	2162 (95.3%)	776 (96.9%)		
	Unmarried	43 (6.2%)	77 (5.7%)	107 (4.7%)	25 (3.1%)		
Maternal occupational status, n (%)						0.872	0.726
	Employed	569 (81.5%)	1111 (82.6%)	1849 (81.7%)	652 (81.4%)		
	Unemployed	129 (18.5%)	234 (17.4%)	414 (18.3%)	149 (18.6%)		
Paternal occupational status, n (%)						0.103	0.024
	Employed	627 (90.5%)	1232 (92.5%)	2090 (93.1%)	739 (93.4%)		
	Unemployed	66 (9.5%)	100 (7.5%)	155 (6.9%)	52 (6.6%)		

Abbreviation: SD: Standard deviation

The association between the six individual healthy lifestyle factors and adolescents’ HRQOL is displayed in Table 2. In crude models, all healthy lifestyle factors were significantly associated with higher HRQOL scores across all dimensions, summary scale, and total scale. After including covariates and mutually adjusting for other healthy lifestyle factors, each healthy lifestyle factor maintained its association with higher HRQOL scores, although the risk estimates were attenuated and some of the associations became statistically non-significant. Specifically, for the HRQOL total scale score, the effect estimates (*β*) of the associations were 4.30 for never smoking (95% CI: 2.16, 6.43), 2.36 for never drinking (95% CI: 1.57, 3.14), 13.93 for good sleep quality (95% CI: 12.99, 14.86), 1.63 for sufficient sleep duration (95% CI: 0.58, 2.67), 8.89 for appropriate Internet use (95% CI: 7.94, 9.84), and 1.67 for adequate physical activity (95% CI: 0.79, 2.56).

**Table 2:** Association between individual healthy lifestyle factor and adolescents’ HRQOL

		*β* (95% CI)					
		Never smoking	Never drinking	Good sleep quality	Sufficient sleep duration	Appropriate Internet use	Adequate physical activity
**Emotional functioning**							
	Crude model	10.37 (6.79, 13.95) *	7.20 (5.94, 8.46) *	26.84 (25.49, 28.18) *	8.15 (6.39, 9.91) *	17.13 (15.63, 18.63) *	3.85 (2.31, 5.40) *
	Adjusted model ^a^	5.08 (1.68, 8.47) *	3.82 (2.57, 5.07) *	22.63 (21.14, 24.12) *	2.83 (1.15, 4.50) *	11.82 (10.31, 13.32) *	1.06 (-0.35, 2.47)
**Social functioning**							
	Crude model	5.63 (3.15, 8.11) *	3.53 (2.65, 4.41) *	12.74 (11.74, 13.74) *	3.71 (2.49, 4.94) *	9.40 (8.34, 10.45) *	1.83 (0.77, 2.90) *
	Adjusted model ^a^	2.12 (-0.51, 4.75)	1.46 (0.50, 2.43) *	11.36 (10.21, 12.51) *	1.20 (-0.09, 2.50)	6.79 (5.62, 7.95) *	1.76 (0.67, 2.85) *
**School functioning**							
	Crude model	13.72 (10.85, 16.58) *	6.84 (5.83, 7.85) *	16.58 (15.43, 17.72) *	4.51 (3.09, 5.94) *	13.30 (12.09, 14.51) *	2.99 (1.74, 4.24) *
	Adjusted model ^a^	8.19 (5.26, 11.12) *	3.20 (2.12, 4.28) *	12.98 (11.70, 14.27) *	1.46 (0.02, 2.91) *	9.82 (8.52, 11.12) *	1.02 (-0.20, 2.24)
**Physical functioning**							
	Crude model	2.50 (0.26, 4.74) *	1.73 (0.94, 2.53) *	10.72 (9.81, 11.63) *	3.10 (2.00, 4.20) *	8.68 (7.73, 9.63) *	4.66 (3.71, 5.61) *
	Adjusted model ^a^	1.79 (-0.49, 4.07)	0.94 (0.11, 1.78) *	8.72 (7.73, 9.72) *	1.01 (-0.11, 2.13)	7.14 (6.13, 8.15) *	2.85 (1.91, 3.79) *
**Psychosocial health summary score**							
	Crude model	9.91 (7.41, 12.41) *	5.86 (4.98, 6.73) *	18.72 (17.78, 19.66) *	5.46 (4.23, 6.69) *	13.28 (12.24, 14.31) *	2.89 (1.81, 3.97) *
	Adjusted model ^a^	5.13 (2.76, 7.50) *	2.83 (1.95, 3.70) *	15.66 (14.62, 16.70) *	1.83 (0.66, 3.00) *	9.47 (8.42, 10.53) *	1.28 (0.30, 2.27) *
**Total scale score**							
	Crude model	8.06 (5.79, 10.32) *	4.83 (4.03, 5.62) *	16.72 (15.86, 17.57) *	4.87 (3.75, 5.98) *	12.13 (11.19, 13.06) *	3.33 (2.36, 4.31) *
	Adjusted model ^a^	4.30 (2.16, 6.43) *	2.36 (1.57, 3.14) *	13.93 (12.99, 14.86) *	1.63 (0.58, 2.67) *	8.89 (7.94, 9.84) *	1.67 (0.79, 2.56) *

Abbreviation: HRQOL, Health-related quality of life; CI, confidence interval

^a^ Adjusted models were controlled for adolescents’ characteristics (age, gender, single-child status, primary caregiver, and boarding school attendance), parental characteristics (age, marital status, and occupational status), and other types of healthy lifestyle factors

Table 3 presents the association between the composite healthy lifestyle groups and HRQOL scores in adolescents. Compared to adolescents with a composite healthy lifestyle score of 0–2, those with scores of 3, 4, or 5–6 had significantly higher HRQOL scores across all dimensions, summary scales, and total scale. These significant associations persisted in the adjusted models. Notably, when compared to adolescents with a composite healthy lifestyle score of 0–2, those reporting 5 or 6 healthy lifestyle factors had significantly higher scores of emotional functioning (*β* = 28.71, 95% CI: 26.69, 30.73), social functioning (*β* = 15.13, 95% CI: 13.62, 16.64), school functioning (*β* = 19.42, 95% CI: 17.73, 21.10), physical functioning (*β* = 12.85, 95% CI: 11.52, 14.18), psychosocial health summary scale (*β* = 21.09, 95% CI: 19.68, 22.49), and total scale (*β* = 19.03, 95% CI: 17.76, 20.30). Specifically, significant dose-response associations were observed between the composite healthy lifestyle groups and the HRQOL scores for all dimensions, summary scales, and total scale in both crude and adjusted models.

**Table 3:** Association between composite healthy lifestyle groups and adolescents’ HRQOL

		*β* (95% CI) by composite healthy lifestyle groups				*P* value for trend
		0–2	3	4	5–6	
**Emotional functioning**						
	Crude model	Ref	14.15 (12.40, 15.90) *	25.67 (24.05, 27.30) *	29.72 (27.78, 31.67) *	< 0.001
	Adjusted model ^a^	Ref	13.82 (12.04, 15.60) *	25.75 (24.07, 27.43) *	28.71 (26.69, 30.73) *	< 0.001
**Social functioning**						
	Crude model	Ref	8.04 (6.75, 9.32) *	13.20 (12.01, 14.40) *	14.86 (13.44, 16.29) *	< 0.001
	Adjusted model ^a^	Ref	7.87 (6.53, 9.21) *	13.35 (12.09, 14.61) *	15.13 (13.62, 16.64) *	< 0.001
**School functioning**						
	Crude model	Ref	9.47 (8.03, 10.91) *	18.43 (17.09, 19.76) *	21.40 (19.80, 23.00) *	< 0.001
	Adjusted model ^a^	Ref	8.75 (7.26, 10.24) *	17.28 (15.88, 18.68) *	19.42 (17.73, 21.10) *	< 0.001
**Physical functioning**						
	Crude model	Ref	5.86 (4.69, 7.02) *	10.50 (9.42, 11.58) *	13.33 (12.04, 14.63) *	< 0.001
	Adjusted model ^a^	Ref	5.82 (4.65, 7.00) *	10.63 (9.53, 11.74) *	12.85 (11.52, 14.18) *	< 0.001
**Psychosocial health summary score**						
	Crude model	Ref	10.55 (9.35, 11.76) *	19.10 (17.98, 20.22) *	21.99 (20.65, 23.33) *	< 0.001
	Adjusted model ^a^	Ref	10.15 (8.90, 11.39) *	18.79 (17.62, 19.96) *	21.09 (19.68, 22.49) *	< 0.001
**Total scale score**						
	Crude model	Ref	9.38 (8.28, 10.47) *	16.95 (15.93, 17.97) *	19.83 (18.61, 21.05) *	< 0.001
	Adjusted model ^a^	Ref	9.07 (7.94, 10.19) *	16.75 (15.70, 17.81) *	19.03 (17.76, 20.30) *	< 0.001

Abbreviation: HRQOL, Health-related quality of life; CI, confidence interval

^a^ Adjusted models were controlled for adolescents’ characteristics (age, gender, single child status, primary caregiver, and boarding school attendance) and parental characteristics (age, marital status, and occupational status). * *P* value < 0.05

The associations of the composite healthy lifestyle groups with adolescents’ HRQOL scores were further evaluated by their genders (Table 4). In both gender groups, significant dose-response associations were observed between the composite healthy lifestyle groups and higher HRQOL scores across all dimensions, summary scales, and total scale (all *P* value for trend < 0.001). We also found that gender was a significant moderator in the associations of the composite healthy lifestyle groups with emotional functioning, school functioning, physical functioning, psychosocial health summary scale, and total scale (all *P* value < 0.05), with girls being more sensitive to healthy lifestyle factors than boys. In contrast, the moderating role of gender in the association between composite healthy lifestyle groups and social functioning was not statistically significant (*P* value = 0.352).

**Table 4:** Gender-specific association between composite healthy lifestyle groups and adolescents’ HRQOL

		*β* (95% CI) by composite healthy lifestyle groups				*P* value for trend	*P* value for interaction	
		0–2	3	4	5–6			
**Emotional functioning**							< 0.001	
	Boys	Ref	13.80 (11.33, 16.28) *	22.43 (20.09, 24.78) *	25.29 (22.62, 27.96) *	< 0.001		
	Girls	Ref	13.67 (11.11, 16.22) *	28.99 (26.60, 31.39) *	32.93 (29.82, 36.04) *	< 0.001		
**Social functioning**							0.352	
	Boys	Ref	9.00 (7.07, 10.94) *	14.00 (12.17, 15.84) *	16.19 (14.10, 18.28) *	< 0.001		
	Girls	Ref	6.75 (4.91, 8.60) *	12.74 (11.01, 14.47) *	14.01 (11.76, 16.25) *	< 0.001		
**School functioning**							0.044	
	Boys	Ref	8.13 (5.98, 10.28) *	15.85 (13.81, 17.89) *	17.97 (15.65, 20.28) *	< 0.001		
	Girls	Ref	9.26 (7.20, 11.32) *	18.62 (16.69, 20.55) *	21.30 (18.79, 23.81) *	< 0.001		
**Physical functioning**							< 0.001	
	Boys	Ref	4.62 (3.09, 6.15) *	8.26 (6.81, 9.71) *	10.12 (8.47, 11.76) *	< 0.001		
	Girls	Ref	6.86 (5.08, 8.65) *	12.90 (11.23, 14.57) *	16.30 (14.13, 18.47) *	< 0.001		
**Psychosocial health summary score**							0.003	
	Boys	Ref	10.31 (8.51, 12.11) *	17.43 (15.72, 19.13) *	19.82 (17.88, 21.75) *	< 0.001		
	Girls	Ref	9.89 (8.17, 11.61) *	20.12 (18.51, 21.73) *	22.75 (20.65, 24.84) *	< 0.001		
**Total scale score**							< 0.001	
	Boys	Ref	8.89 (7.29, 10.48) *	15.14 (13.62, 16.65) *	17.39 (15.67, 19.11) *	< 0.001		
	Girls	Ref	9.14 (7.56, 10.71) *	18.31 (16.84, 19.79) *	21.13 (19.22, 23.05) *	< 0.001		

Abbreviation: HRQOL, Health-related quality of life; CI, confidence interval

Models were controlled for adolescents’ characteristics (age, single child status, primary caregiver, and boarding school attendance) and parental characteristics (age, marital status, and occupational status). * *P* value < 0.05

## Discussion

In this cross-sectional study, we found that healthy lifestyle factors, including never smoking, never drinking, good sleep quality, sufficient sleep duration, appropriate Internet use, and adequate physical activity, were independently associated with better HRQOL in adolescents. In addition, the composite healthy lifestyle scores showed a dose-response pattern with HRQOL across all dimensions, summary scales, and total scale. Gender was a significant moderator in the association between the composite healthy lifestyle scores and different HRQOL dimensions, summary scales, and total scale, with the exception of the social functioning dimension. Girls were found to be more sensitive to healthy lifestyle factors than boys.

Our findings of the positive associations between composite healthy lifestyle scores and adolescents’ HRQOL were in line with previous studies conducted in European counties [24, 25]. One study in Spain has considered five healthy lifestyle factors, including physical activity, adherence to the Mediterranean diet, sleep quality, sleep duration, and screen time [24]. The results indicated that the number of healthy lifestyle factors was positively associated with HRQOL scores measured by the KIDSCREEN-10 questionnaire [24]. Another cross-sectional study of 5024 adolescents has included healthy lifestyle factors of physical activity, screen-based time, sleep duration, fruit and vegetable consumption, drinking, and smoking, and demonstrated a significant association of the composite healthy lifestyle measure with better self-rated health and higher HRQOL [25]. Similar findings have also been reported in studies focusing on other outcomes in children and adolescents [43, 44]. For example, a cross-sectional study has found that a lower number of healthy lifestyle factors was associated with higher risks of depressive symptoms among Chinese adolescents [43]. Moreover, a nationwide cross-sectional study in China has shown that compared with adolescents who had a healthy lifestyle, those with an unhealthy lifestyle had a higher risk of obesity [44].

The mechanisms underlying the positive association between healthy lifestyle factors and HRQOL scores are not yet fully understood. Several possible explanations might account for such associations. First, previous studies have indicated that healthy lifestyle factors could reduce chronic inflammatory levels, subsequently leading to improved HRQOL [45–47]. Second, unhealthy lifestyle factors such as sleep problems and Internet addiction have been shown to be associated with stress and negative emotions, which might stimulate the hypothalamic-pituitary-adrenal axis, increase cortisol levels, and ultimately lead to mental problems in adolescents, resulting in worse HRQOL [48–52]. Conversely, healthy lifestyle factors can help maintain better HRQOL among adolescents. Third, appropriate Internet use is beneficial to facilitate technical skills, social connection, and communication in adolescents, thereby promoting their mental well-being and ultimately better HRQOL [53, 54]. Therefore, the significant association between healthy lifestyle factors and better HRQOL was plausible.

Our study also found a moderating role of gender in the association between cumulative healthy lifestyle scores and adolescents’ HRQOL across all dimensions, except for social functioning. Although there are limited similar studies to compare with, this finding aligns with previous studies that have identified gender-specific associations between healthy lifestyle factors and other health-related outcomes in adolescents [43, 55]. For example, a cross-sectional study among 3967 Chinese adolescents aged 11–19 years has observed a stronger association between the number of unhealthy lifestyle factors and depressive symptoms in girls compared to boys [43]. Similarly, research conducted in India has also found a link between cumulative unhealthy lifestyle factors and obesity in girls, while boys only exhibited an increased BMI [55]. However, contradictory findings were reported in another study in China, which has shown a relatively larger effect size in boys compared to girls regarding the association between reduced weekend sleep duration and abdominal obesity [56]. One possible explanation of the discrepancies might be attributed to the diverse healthy lifestyle factors investigated across different studies. In addition, while most prior studies focused on a specific health outcome, our study employed a multidimensional construct that could reflect the overall self-rated health. Nevertheless, further studies are still needed to explore the possible gender-specific association between the combined healthy lifestyle factors and HRQOL in young populations, and to uncover the possible underlying mechanisms.

Our study benefited from a large sample size, which allowed us to conduct analyses with sufficient statistical power. By using HRQOL as the outcome, we were able to assess the overall health of the young population. In addition, we assessed not only the impact of individual healthy lifestyle factors, but also the combined impact of these healthy lifestyle factors on adolescents’ HRQOL. We also evaluated the moderating role of gender in this association to identify sensitive populations. Nevertheless, several limitations should be acknowledged as well. First, due to the cross-sectional nature of the study, we were unable to establish a causal relationship between the exposure and the outcome. Future longitudinal studies are needed to better understand the temporal association between healthy lifestyle factors and adolescents’ HRQOL. Second, our participants were recruited exclusively from one megacity. The generalizability of the findings should be considered with caution. Third, although a healthy diet has been recognized as a critical healthy lifestyle factor associated with HRQOL in adolescents [57], we did not include it in our study due to data unavailability. Fourth, we utilized the composite healthy lifestyle scores in our analyses, assuming each lifestyle factor equally impacts HRQOL. While this approach might not reflect the real-life situation, it is straightforward and can be easily understood by adolescents during preventive interventions. Furthermore, we have assessed the associations with consideration of different weighting for each lifestyle factor in sensitivity analysis (data not shown) and the findings were consistent, indicating the reliability of our conclusions. Last, while several confounders were controlled in the multivariate analyses, unmeasured confounders and modifiers may still be present [58–60].

## Conclusions

In conclusion, we found that adherence to an overall healthy lifestyle, including never smoking, never drinking, good sleep quality, sufficient sleep duration, appropriate Internet use, and adequate physical activity, was associated with higher HRQOL scores in adolescents. In addition, the associations tended to be stronger in girls. These findings implied that health education and public health interventions aimed at promoting healthy lifestyle factors in adolescents may be an effective strategy for improving their HRQOL, especially among girls. However, further randomized controlled trials are needed to confirm the conclusion.

## Data Availability

The datasets used and analyzed during the current study are not publicly available for ethical and privacy reasons but are available from the corresponding author upon reasonable request.
